# Interaction between carbon metabolism and phosphate accumulation is revealed by a mutation of a cellulose synthase-like protein, CSLF6

**DOI:** 10.1093/jxb/erv050

**Published:** 2015-03-04

**Authors:** Cheng Jin, Chuanying Fang, Hui Yuan, Shouchuang Wang, Yangyang Wu, Xianqing Liu, Yuanyuan Zhang, Jie Luo

**Affiliations:** ^1^National Key Laboratory of Crop Genetic Improvement and National Center of Plant Gene Research (Wuhan), Huazhong Agricultural University, Wuhan 430070, China; ^2^College of Life Science and Technology, Huazhong Agricultural University, Wuhan 430070, China

**Keywords:** Cellulose, phosphate (Pi) accumulation, Pi transporters, sucrose.

## Abstract

Phosphorus is an essential macronutrient for plant growth and development. However, the network that affects phosphate (Pi) accumulation in crops is not well established. It is reported here that OsCSLF6, a member of the cellulose synthase-like family (CSLF), which is found only in grasses, is involved in Pi accumulation. The *oscslf6* mutants (*oscslf6-1* and *oscslf6-2*) display Pi toxic symptoms and increased Pi accumulation in both roots and shoots under the Pi-sufficient condition, which correlate with the induced expression of Pi transporters in the knockout mutants. Consistent with the over-accumulation of Pi, a significant decrease in primary root length, adventitious root length, and adventitious root number were observed in the *oscslf6* mutants when compared with the wild type (WT) under Pi-sufficient conditions. In addition, the sucrose (Suc) level was increased in the *oscslf6* mutants and the expression of sucrose synthases *(OsSUS4/5*) and sucrose transporters (*OsSUT1/2/4/OsSweet14*) genes were also induced in the shoots of *oscslf6* mutants, suggesting that *OsCSLF6* may play a role in affecting Pi accumulation by affecting the level of carbon metabolism.

## Highlight

*OsCSLF6* plays an important role in balance carbon metabolism and phosphate accumulation.

## Introduction

Phosphorus is an essential macronutrient for plant growth, development, and reproduction. Despite the abundance of Pi in the soil, the form of Pi available for uptake by plants is usually present at a low level in the soil due to its precipitation with cations and conversion to organic matter (Kuo and Chiou, 2011). To cope with heterogeneous or low phosphate (Pi) availability, plants have evolved complex adaptive responses that include morphological and physiological modifications. These responses include changes in root architecture and morphology, increased Pi uptake activity, the secretion of organic acids or phosphatase, and an association with mycorrhizal fungi (López-Bucio *et al.*, 2003; Aung *et al.*, 2006; Chiou and Lin, 2011; Péret *et al.*, 2011).

In recent decades, a number of genes that are involved in sensing and responding to Pi deficiency, and in their regulatory networks, have been isolated from vascular plants, including Pi transporters, phosphatases, and RNases. In *Arabidopsis*, a major regulatory system that involves SPX1, *PHR1*, *SIZ1*, *miR399*, and *PHO2* in response to Pi deficiency has been identified (Fujii *et al.*, 2005; Miura *et al.*, 2005; Aung *et al.*, 2006; Chiou *et al.*, 2006; Nilsson *et al.*, 2007; Puga *et al.*, 2014). MYB transcription factors *AtPHR1* and *AtPHL1* have been identified as key regulators in the Pi-signalling pathway (Rubio *et al.*, 2001; Bustos *et al.*, 2010). Over-expression of *AtPHR1* leads to the over accumulation of Pi in shoots and the activation of Pi starvation-induced gene expression (Nilsson *et al.*, 2007). Puga *et al.* (2014) have reported that the nuclear protein SPX1 is a Pi-dependent inhibitor of PHR1 in *Arabidopsis*. *SIZ1*, a SUMO E3 ligase, is known to control *PHR1* sumoylation (Miura *et al.*, 2005). Upon Pi starvation, *miR399* is up-regulated by *PHR1* and is involved in the cleavage of the PHO2 mRNA, which encodes the low-Pi-responsive UBC24 (ubiquitin-conjugating E2 24) enzyme (Aung *et al.*, 2006; Bari *et al.*, 2006; Chiou *et al.*, 2006). Both *miR399* over-expression and mutations in *PHO2* resulted in the over-accumulation of Pi and Pi toxic symptoms being exhibited in *Arabidopsis* (Fujii *et al.*, 2005; Aung *et al.*, 2006; Chiou *et al.*, 2006).

The functional orthologue of *AtPHR1* in rice (designated as *OsPHR2*) was also identified, and it was found that over-expression of *OsPHR2* resulted in the excessive accumulation of Pi in shoots and the up-regulation of some Pi transporter genes under Pi-sufficient conditions (Zhou *et al.*, 2008a; Liu *et al.*, 2010). OsSPX4 has been reported to function in Pi starvation signalling and to act as a negative regulator of *OsPHR2* in rice (Lv *et al.*, 2014). OsSPX1 and OsSPX2 inhibit phosphate starvation responses through interacting with PHR2 in a phosphate-dependent manner (Wang *et al.*, 2014). *OsPHO2*, the putative homologue of *AtPHO2*, has been shown to be involved in the Pi starvation signalling pathway mediated by *OsSPX1–OsPHR2* (Bari *et al.*, 2006; Wang *et al.*, 2009; Liu *et al.*, 2010).

Although our understanding of Pi starvation signalling involving *SPX4*, *SPX1*, SPX2, *PHR2*, *miR399*, and *PHO2* is well established, other pathways may also be required for the Pi starvation response (PSR). For example, characterization of transcription factors such as *OsPTF1* (Yi *et al.*, 2005), *OsMYB2P-1* (Dai *et al.*, 2012), *AtWRKY75* (Devaiah *et al.*, 2007a), *AtZAT6* (Devaiah *et al.*, 2007b), *AtMYB62* (Devaiah *et al.*, 2009), *AtBHLH32* (Chen *et al.*, 2007), *AtWRKY6* (Chen *et al.*, 2009), and *AtMYB2* (Baek *et al.*, 2013) suggests that they play crucial roles in controlling the expression of the downstream genes as well as the regulation of cross-talk among different signalling pathways.

Besides these factors, sugar signalling also plays important roles in regulating plant responses to Pi starvation. Many studies have shown the importance of sugar signalling in regulating PSR, including the increased expression of PSI genes and changes in root system architecture (RSA) (Liu *et al.*, 2005; Müller *et al.*, 2005, 2007; Jain *et al.*, 2007; Karthikeyan *et al.*, 2007; Hammond and White, 2008; Zhou *et al.*, 2008b; Chiou and Lin, 2011). Increased Suc concentrations in roots precede the induction of PSR and the inhibition of Suc biosynthesis or translocation attenuates the plant response to Pi starvation (Hammond and White, 2008, 2011). However, cross-talk between carbon metabolism and the Pi pathway in rice remains unclear.

A rice mutant that displayed Pi toxicity symptoms was identified here. Sequencing of the flanking regions of the T-DNA insertion site revealed that the insertion leads to the loss-of-function of the *OsCSLF6,* a gene encoding a cellulose synthase-like protein. The increased Pi content in *oscslf6* mutants was associated with the increased expression of PHT genes. *OsCSLF6* was also involved in Pi-dependent root architecture alteration. Moreover, the Suc level was increased and the expression of genes encoding sucrose synthases (*OsSUS4/5*) and sucrose transporters (Os*SUT1/2/4/Sweet14*) were induced in shoot of *oscslf6* mutants, suggesting that *OsCSLF6* may affect Pi accumulation and response through the alteration of carbon metabolism in rice.

## Materials and methods

### Isolation of T-DNA insertion mutants

The mutant line 04Z11DM89 [*oscslf6-1*; rice (*Oryza sativa* ssp. *japonica* cv. Zhonghua 11)] was obtained from the RMD database (Wu *et al.*, 2003; Zhang *et al.*, 2006) (http://rmd.ncpgr.cn/) and 3A-60123 (*oscslf6-2*; rice ssp. *japonica* cv. Dongjin) was ordered from the POSTECH RISD database (Jeon *et al.*, 2000) (http://www.postech.ac.kr/life/pfg/risd/), respectively. Mutants were planted in the paddy field of Huazhong Agricultural University in the normal rice (*Oryza sativa*) growing season of Wuhan, China, and in a greenhouse during the winter. All transgenic plants were grown in similar growth conditions.

### Hydroponic experiments

Hydroponic experiments were conducted using normal rice culture solution with 10mg l^–1^ Pi (Yoshida *et al.*, 1976) and a Pi-deficient solution (0.5mg l^–1^ Pi). Rice seeds were surface-sterilized for 10min with ethanol (75% v/v) and for 15min with commercially diluted (1:3, v/v) NaClO, washed, and germinated for 3 d at 28 °C. The 9-d-old seedlings were transferred to nutrient solution containing 1.25mM NH_4_NO_3_, 0.35mM K_2_SO_4_, 1mM CaCl_2_.2H_2_O, 1mM MgSO_4_.7H_2_O, 0.5mM Na_2_SiO_3_.9H_2_O, 20mM Fe-EDTA, 20mM H_3_BO_3_, 9mM MnCl_2_.4H_2_O, 0.32mM CuSO_4_.5H_2_O, 0.77mM ZnSO_4_.7H_2_O, and 0.39mM Na_2_MoO_4_.2H_2_O, pH 5.5, supplemented with 10mg l^–1^ Pi (HP) or 0.5mg l^–1^ Pi (LP); 30-d-old seedlings were observed for phenotype or sampled for total P concentration measurement (Zhou *et al.*, 2008a). The solution was refreshed every 3 d.

### Vector construction and plant transformation

An 11kb genomic DNA fragment containing the entire *OsCSLF6* coding region and the 2756bp upstream and 2818bp downstream sequences was isolated by digestion of the Clemson BAC clone OSJNB*a0055L06* (kindly provided by R Wing, University of Arizona) and inserted into the binary vector pCAMBIA2301. An empty pCAMBIA2301 vector was used as a control. The transformation recipient was callus culture that was induced from seeds homozygous for *cslf6-1.*

To fuse the Os08g06380 promoter to the GUS gene, the promoter of *OsCSLF6*, a 1878bp fragment upstream of the ATG of Os08g06380 was amplified by PCR. The PCR product was cloned into pDONR207 by BP recombination. After sequencing, the correct clone for each gene was individually introduced into the Gateway-compatible GUS fusion vector pGWB3 (Nakagawa *et al.*, 2007) to produce CSLF6pGUS.

All the constructs were introduced into *Agrobacterium tumefaciens* EHA105 and were transformed into the callus derived from the *japonica* cultivar Zhonghua 11 by *Agrobacterium*-mediated transformation as described previously (Wu *et al.*, 2003). All primers for genotyping and vector construction are listed in Supplementary Table S1 at *JXB* online.

### Scanning electron microscopy

Samples were prepared according to the method previously reported by Mou *et al.* (2000), with some modifications. In brief, the mature culms were excised with a blade and immediately placed in 70% ethanol, 5% acetic acid, and 3.7% formaldehyde for 24h. Then samples were critical-point dried, sputter-coated with gold, and observed with a scanning electron microscope (S570, Hitachi, Tokyo, Japan).

### Histochemical staining

Cellulose staining was assayed according to the method described by Li *et al.* (2003). Fresh hand-cut sections (~20 μm thick) from rice culms were stained with a 0.005% aqueous solution of Calcofluor (fluorescent brightener 28; Sigma) for 2min and visualized with a fluorescent microscope (Leica, Wetzlar, Germany).

### GUS staining

GUS staining was performed as previously described by Jefferson *et al.* (1987). Samples were transferred to a solution of 200mM sodium phosphate buffer, pH 7.0, 12.5mM potassium ferricyanide, 12.5mM potassium ferrocyanide, 0.3% Triton X-100, 20% methanol, and 38.3mM 5-bromo-4-chloro-3-indolyl-β-D-glucuronide and were kept overnight at 37 °C. The stained samples were then washed with 75% ethanol overnight. The cleared samples were observed by light microscopy.

### Measurement of total Pi

Dry samples (approximately 0.2g) were used for the determination of total Pi as previously described by Zhou *et al.* (2008a).

The Pi uptake rate was measured based on the rate of depletion of the nutrient from solution over 24h (Liu *et al.*, 2010). Thirty-day-old plants were used. Before measurement, the plants were moved into a solution culture without Pi for 3 d and then transferred to a pot with four plants per litre of fresh solution (10mg l^–1^ Pi). A 1ml aliquot of solution was removed from each pot at 24, 48, and 72h time points for phosphorus concentration analysis by the phosphomolybdenum blue reaction. The roots of the plants in each pot were harvested and oven-dried, and the Pi uptake rate was calculated as depletion of the Pi in the solution per gram of dried root biomass.

### RT-PCR and real-time PCR

Total RNA was extracted from rice using an RNA extraction kit (TRIzol reagent; Invitrogen) according to the manufacturer’s instructions. The first-strand cDNA was synthesized using 3 μg of RNA and 200U of M-MLV reverse transcriptase (Invitrogen) according to the manufacturer’s protocol. Real-time PCR was performed using an optical 96-well plate in an ABI Stepone plus PCR system (Applied Biosystems) by using SYBR Premix reagent F-415 (Thermo Scientific). The expression measurements were obtained using the relative quantification method (Livak and Schmittgen, 2001). All the primers used for RT-PCR and real-time PCR are listed in Supplementary Tables S1 and S2 at *JXB* online.

### Quantitative analysis of sugar contents

Twelve-day-old seedlings were cut into shoot and root parts and thoroughly ground in liquid nitrogen. Soluble sugar was extracted in 225 μl methanol, 120 μl CHCl_3_, and 240 μl ddH_2_O at 70 °C for 15min. Samples were centrifuged at 12 000rpm for 10min, transferred to 200 μl supernatant and then dried at 80 °C. For methoximation, 40 μl of methoxyamine hydrochloride in pyridine (20mg ml^–1^) was added at 30 °C for 90min. After, 60 μl of *N*-methyl-*N*-trimethylsily-trifluoroacetamide was added, and the mixture was incubated at 37 °C for 30min. The derivatives were analysed by gas chromatography–mass spectrometry on a Thermo DSQII mass spectrometer using a DB-5ms column. A temperature programme was implemented as follows: initially at 70 °C, followed by heating to 300 °C at 5 °C min^–1^, and then held at 300 °C for a further 3min. Myoinositol was used as an internal standard.

## Results

### Isolation and characterization of the *oscslf6* mutants in rice

**Fig. 1.**
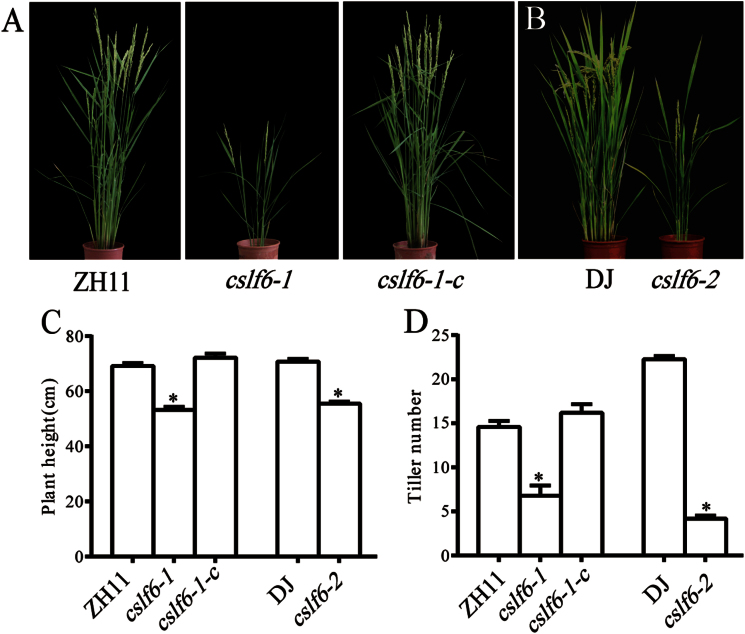
Phenotypes of wild type (WT) and *oscslf6* mutants grown in the field for 3 months. (A) The phenotypes of ZH11 (Zhonghua11) and *oscslf6-1* and *cslf6-1*-complemented (*cslf6-1-c*) mutant plants at maturity. (B) The phenotypes of DJ (Dongjin) and *oscslf6-2* mutant plants at maturity. (C) Height of ZH11, *oscslf6-1*, *cslf6-1*-*c*, DJ, and *oscslf6-2* mutant plants. (D) Tiller number of ZH11, *oscslf6-1*, *cslf6-1*-*c*, DJ, and *oscslf6-2* mutant plants. Error bars indicate SD (*n*=10). Asterisks indicate the significance of differences between WT and *cslf6* mutant plants as determined by Student’s *t* test: *P ≤0.05. (This figure is available in colour at *JXB* online.)

A rice mutant with greatly reduced height and tiller number was isolated in screening transgene lines after *Agrobacterium tumifaciens*-mediated T-DNA insertion (Wu *et al.*, 2003; Zhang *et al.*, 2006). Sequencing of the flanking regions of the T-DNA insertion site indicated that the T-DNA was inserted in the first intron of *OsCSLF6* (see Supplementary Fig. 1A at *JXB* online). The mutant, designated as *oscslf6-1*, exhibited dwarfism throughout growth and development (Fig. 1A). At the mature stage, the height of mutant plants was about 70% of that of the WT (ZH11) plants (Fig. 1C). In addition to dwarfism, *oscslf6-1* showed a marked decrease in tiller number (Fig. 1D). The Rice Functional Genomic Express database (http://signal.salk.edu/cgi-bin/RiceGE) was searched using the *OsCSLF6* sequence and an allelic mutant PFG_3A-60123.L, was found which was named *oscslf6-2.* The T-DNA insertion site was located at 204bp upstream of ATG of LOC_Os08g06380 (see Supplementary Fig. 1A at *JXB* online). The *Oscslf6-2* mutant also displayed similar phenotypes including reduced plant height and a decrease in tiller number (Fig. 1B–D).

Reverse transcription-PCR analysis indicated that *oscslf6-1* and *oscslf6-2* are knockout lines since no *OsCSLF6* transcript could be detected in plants homozygous for the insertions (see Supplementary Fig. 1B at *JXB* online). To verify further that the mutant phenotype is the result of the loss-of-function of *OsCSLF6*, an 11kb *Bam*H1–*Bam*H1 fragment harbouring the entire *OsCSLF6* coding region, a 2.7kb upstream and a 2.8kb downstream region were introduced into the *oscslf6-1* mutant background. As expected, tillers and height defects of the complemented *oscslf6-1* (*cslf6-1*-c) mutant were rescued (Fig. 1A–D).

### The *oscslf6* mutants display overaccumulation of Pi

**Fig. 2.**
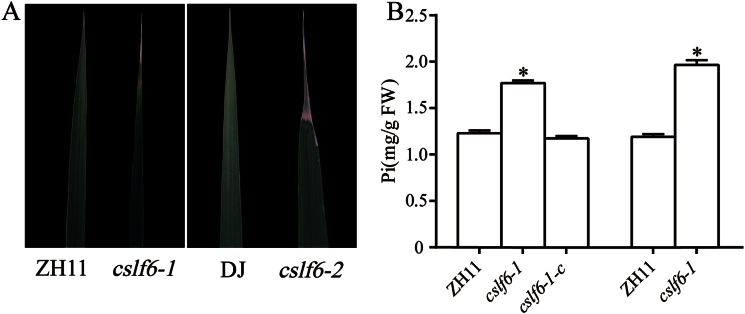
Pi content in shoots of *oscslf6* mutants. (A) Leaf tip necrosis of WT and *oscslf6* mutants plants grown in the field for 3 months. (B) Shoot Pi content in 3-month-old field-grown ZH11, *oscslf6-1*, *cslf6-1*-*c*, DJ, and *oscslf6-2* mutant plants. Error bars indicate SD (*n*=10). Asterisks indicate the significance of differences between WT and *oscslf6* mutant plants as determined by Student’s *t* test: *P ≤0.05. (This figure is available in colour at *JXB* online.)

Necrotic spots were observed in the mature leaves of *oscslf6-1* and *oscslf6-2* plants in Pi-sufficient soil (Fig. 2A). The leaf toxic symptoms and growth retardation of the *oscslf6* mutants are similar to that of Pi over-accumulation plants such as *OsPHR2*-overexpression plants (*OsPHR2(O)*) and *ospho2* mutants (Zhou *et al.*, 2008a; Hu *et al.*, 2011).

To explore whether the phenotype of the *oscslf6* mutants is related to Pi overaccumulation, Pi concentrations in the shoots of the *oscslf6* and *cslf6-1*-*c* plants were examined in Pi-sufficient soil. The results indicated Pi concentrations in shoots of *oscslf6-1* and *oscslf6-2* mutant plants were significantly higher than those of WT plants (Fig. 2B). The Pi concentrations of *cslf6-1*-*c* plants were rescued to the level of their WT counterparts (Fig. 2B). To determine whether an excess of Pi was responsible for the leaf toxic symptoms and growth retardation phenotype of *oscslf6* mutants, *oscslf6*, *cslf6*-*1-c*, and WT plants were grown in nutrient solutions that were supplied with either a high level of Pi (HP, 0.323mM) or a low level of Pi (LP, 0.032mM) for phenotype observation.

**Fig. 3.**
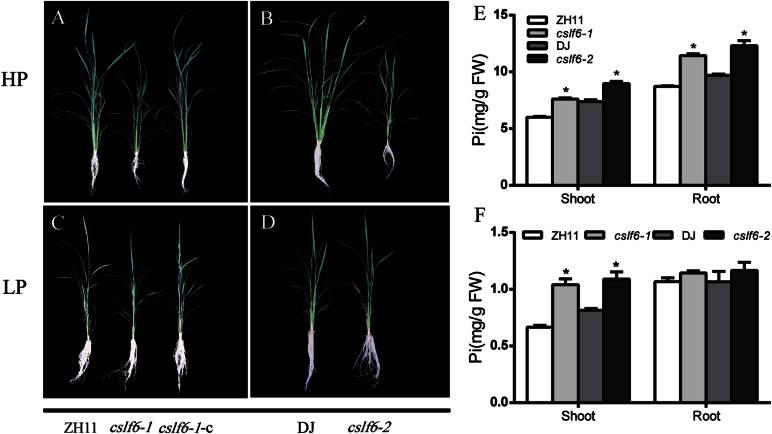
Phenotypes of *oscslf6* mutant plants under HP and LP conditions. (A) Phenotype of WT (ZH11), *oscslf6-1* mutant, and *cslf6-1-c* plants after being grown under HP conditions (0.3mM Pi) for 30 d. The Pi toxic phenotype shows a smaller plant, reduced tiller number, and decreased shoot and root length. (B) Phenotype of WT (DJ) and *oscslf6-2* mutants after being grown under HP conditions (0.3mM Pi) for 30 d. (C) Phenotype of WT (ZH11), *oscslf6-1*, and *cslf6-1*-*c* plants after being grown under LP conditions (0.015mM Pi) for 30 d. (D) Phenotype of the wild type (DJ) and *oscslf6-2* mutants after being grown under LP conditions (0.015mM Pi) for 30 d. (E) Pi contents in shoots and roots of the wild type (ZH11), *oscslf6-1*, *cslf6-1*-*c*, DJ, and *oscslf6-2* mutant plants under Pi-sufficient conditions. (F) Pi contents in shoots and roots of WT (ZH11), *oscslf6-1*, *cslf6-*1-*c*, WT (DJ), and *oscslf6-2* mutant plants under Pi-deficient conditions. Plants were pregerminated in water for 7 d and then grown hydroponically for 30 d under HP and LP conditions. Error bars indicate SD (*n*=10). Asterisks indicate the significance of differences between WT and *oscslf6* mutant plants as determined by Student’s *t* test: *P ≤0.05. DW, dry weight. (This figure is available in colour at *JXB* online.)

In the HP condition, both knockout lines of *oscslf6* (*oscslf6-1* and *oscslf6-2*) displayed toxic leaf symptoms and growth retardation similar to that of the *OsPHR2*-overexpressing plants [*PHR2*(*O*)], the *OsPT2*-overexpressing plants [*OsPT2*(*O*)], and the rice *ltn1* (*ospho2*) mutant plants (Fig. 3A, B) (Zhou *et al.*, 2008a; Wang *et al.*, 2009; Liu *et al.*, 2010; Hu *et al.*, 2011), as indicated by small plants, reduced tiller number, and decreased tiller number and decreased shoot and root biomass of the *oscslf6* mutant lines (Fig. 3A–D; Table 1). Moreover, Pi concentrations in both shoots and roots of the *oscslf6* mutant plants grown in HP conditions were higher than those in the WT plants (Fig. 3E).

**Table 1.** Plant height, tiller number, dry shoot biomass, and dry root biomass of WT (ZH11 and DJ), cslf6-1-complemented and oscslf6 mutants The 9-d-old seedlings were transferred to HP or LP medium for 30 d and then the plants were sampled for the measurements. The values are means ±SD of three independent experiments, with 10 seedlings being used in each experiment. Asterisks indicate the significance of differences between the wild type and *oscslf6* mutant plants as determined by Student’s *t* test analysis: *0.01≤ *P* ≤0.05, **P <0.01.

Genotype	Plant height (cm)	Tillering number	Shoot biomass (g DW)	Root biomass (g DW)
**HP**				
ZH11	73.533±1.784	8.167±0.307	1.660±0.137	0.327±0.030
*cslf6-1*	59.117±1.282**	4.333±0.211**	0.784±0.042**	0.180±0.013**
*cslf6-1-c*	74.433±0.914	7.500±0.224	1.636±0.026	0.340±0.010
DJ	68.433±0.878	7.400±0.245	1.604±0.077	0.340±0.014
*cslf6-2*	53.200±0.683**	3.400±0.245**	0.556±0.043**	0.168±0.017**
**LP**				
ZH11	51.867±0.630	3.167±0.307	0.652±0.026	0.294±0.010
*cslf6-1*	42.133±0.455**	2.833±0.167*	0.455±0.014*	0.220±0.013**
*cslf6-1-c*	51.700±0.599	3.667±0.211	0.749±0.026	0.320±0.004
DJ	44.817±0.549	3.000±0.000	0.357±0.008	0.239±0.012
*cslf6-2*	42.850±0.809	2.833±0.167	0.363±0.011	0.224±0.011

The tiller number, shoot and root biomass of *oscslf6-1* mutants decreased under Pi-sufficient conditions and this was alleviated under Pi-deficient conditions (Fig. 3C, D; Table 1). Similarly, those parameters in *oscslf6-2* mutant were recovered to almost the same levels as in WT plants (Fig. 3C, D; Table 1). Furthermore, leaf necrosis in the *oscslf6* mutants wasnot seen when those plants were grown under Pi-deficient conditions. In LP conditions, higher Pi concentrations in the shoots of *oscslf6-1* and *oscslf6-2* mutants were also observed, whereas, in roots, the Pi concentration was similar to the WT (Fig. 3F) although the absolute concentration level was much lower than that at the HP level.

To confirm further that excessive Pi accumulation of *oscslf6* mutants is indeed caused by the loss-of-function of *OsCSLF6, cslf6-1*-*c* lines were also investigated using 30-d-old plants grown under both HP and LP conditions (Fig. 3A, C; Table 1). The results showed the Pi concentration and growth retardation of *cslf6-1-c* plants were almost rescued to the levels of the WT plants, suggesting strongly that phenotypes of *oscslf6-1* mutants are caused by the loss-of-function of *OsCSLF6* (Fig. 3A, C; Table 1).

To explore whether the phenotype of *oscslf6* mutants is related to nitrogen (N) accumulation, N concentrations in the shoots of the *oscslf6* plants were also examined. The result showed that the N content was slightly increased in the shoots of the *oscslf6-1* mutants but was not changed in the *oscslf6-2* mutants compared with the WT (see Supplementary Fig. 2A at *JXB* online). However, expression of most nitrate transporters and ammonium transporters were not significantly changed in the shoots of *oscslf6* mutants (see Supplementary Fig. 2B at *JXB* online). Furthermore, no alteration of the phenotypes was found in mutants under different N supply conditions (Supplementary Fig. 2C at *JXB* online). The phenotypes of toxic leaf symptoms and growth retardation were still observed in *oscslf6-1* mutants under high-nitrogen (+10N;12.5mM NH_4_NO_3_) and N-deficient (no NH_4_NO_3_) conditions (see Supplementary Fig. 2C at *JXB* online). These results suggested that nitrogen was not the reason for the phenotypes of *oscslf6* mutants.

Taken together, these results indicate that *OsCSLF6* is involved in the regulation of shoot and root Pi accumulation.

### Knockout of *OsCSLF6* affects development of primary and adventitious roots

**Fig. 4.**
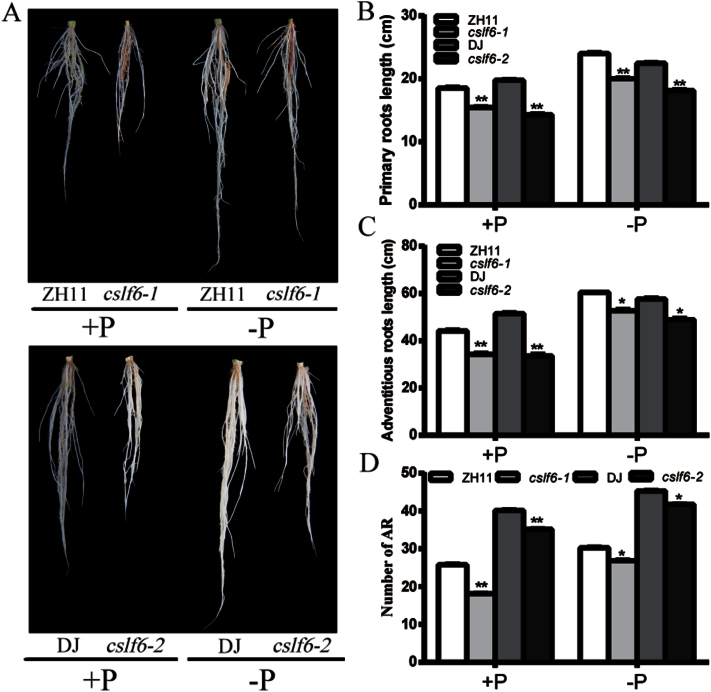
Effects of Pi availability in the medium on root architecture in WT (ZH11), *oscslf6-1*, WT (DJ), and *oscslf6-2* mutant plants. (A) Root performance of 14-d-old seedlings of WT (ZH11), *oscslf6-1*, WT (DJ), and *oscslf6-2* mutant plants under Pi-sufficient and Pi-deficient conditions. (B–D) The length of primary roots (B), the three longest adventitious roots (C), and the number of adventitious roots (AR) (D) of WT (ZH11), *oscslf6-1*, WT (DJ), and *oscslf6-2* mutant plants under Pi-sufficient or Pi-deficient conditions. Error bars indicate SD (*n*=10). Asterisks indicate the significance of differences between WT and *cslf6* mutant plants as determined by Student’s *t* test: *0.01≤ *P* ≤0.05, **P <0.01.

A suite of studies have shown that plants adjust their root structure and morphology to increased Pi availability (Niu *et al.*, 2013). The root architecture system is sensitive to Pi starvation, as indicated by the elongation of primary and adventitious roots in rice (Wissuwa, 2003; Yi *et al.*, 2005). To test whether *OsCSLF6* is involved in root architecture alteration in response to Pi concentration, plants grown under Pi-sufficient or Pi-deficient hydroponic culture conditions for 14 d were used to compare the primary root length, adventitious root (AR) number, and total length of the three longest adventitious roots (Fig. 4A). Under Pi-sufficient conditions, primary and adventitious roots in the *oscslf6* mutants were shorter than those in wild-type plants (Fig. 4A–C). There was a significant decrease in the number of adventitious roots of the mutant under both Pi-sufficient and Pi-deficient conditions compared with the WT (Fig. 4D). The primary roots, adventitious roots, and adventitious root number were more highly induced in mutants than in the WT under the LP condition (Fig. 4A–D). Consistently, Pi contents in *oscslf6* roots were also rescued to the WT level under Pi starvation (Fig. 3F). Taken together, these results suggested that *OsCSLF6* is involved in Pi-dependent root architecture alteration possibly by affecting the Pi contents in roots.

### Knockout of *OsCSLF6* affects the expression of Pi transporters

**Fig. 5.**
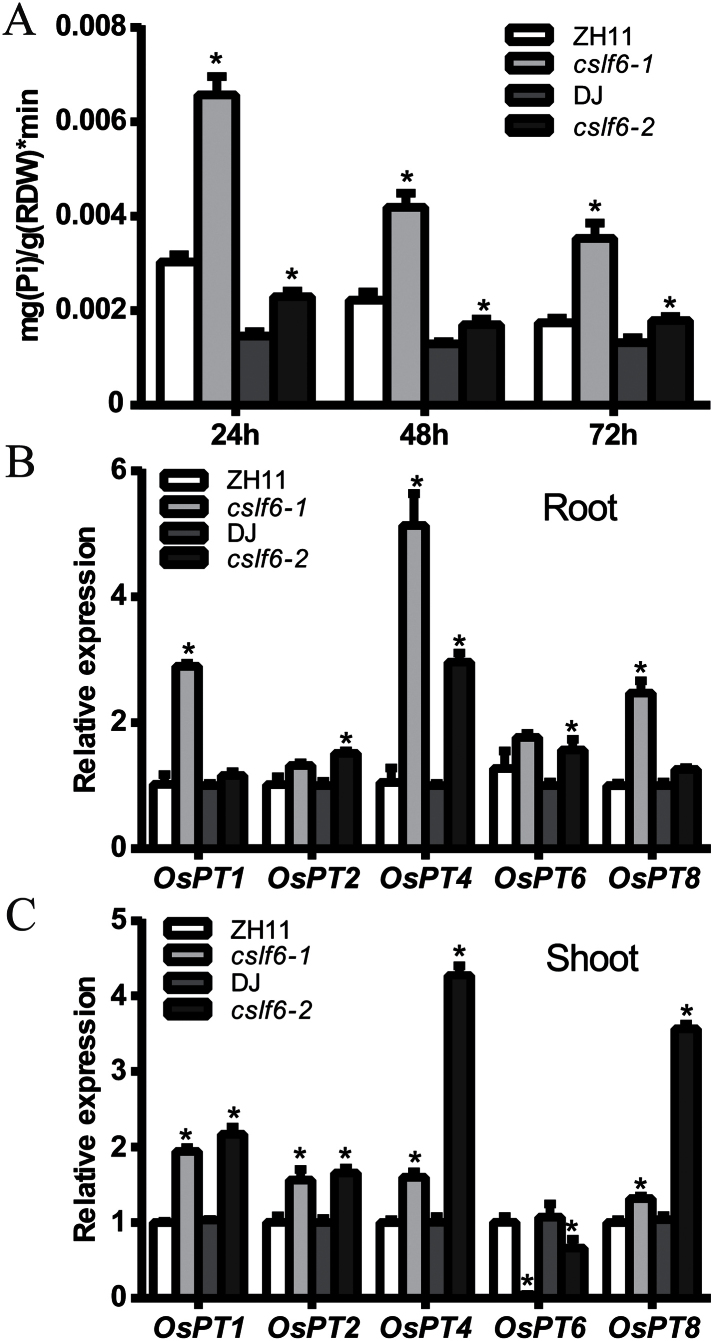
Pi uptake of WT (ZH11), *cslf6-1*, WT (DJ), and *oscslf6-2* mutant plants. (A) Pi uptake of WT (ZH11), *oscslf6-1*, WT (DJ), and *oscslf6-2* mutant plants under HP conditions. (B–C) Expression of Pi transporter genes in roots (B) and shoots (C) of WT (ZH11 and DJ) and *oscslf6* mutant plants under HP conditions. Expression was normalized to that of ubiquitin. Error bars indicate SD (*n*=3). Asterisks indicate the significance of differences between WT and *oscslf6* mutant plants as determined by Student’s *t* test: *P ≤0.05.

A Pi uptake experiment was then performed to explore whether Pi over-accumulation in *oscslf6* was due to increased Pi uptake. The Pi uptake rates of *oscslf6* mutants were significantly higher than that of WT at 24, 48, and 72h (Fig. 5A). The result indicates that knockout of *OsCSLF6* leads to an increase in Pi uptake, which may result in the accumulation of excess Pi under abundant Pi conditions.

A number of putative high-affinity Pi transporter genes that function in Pi uptake, translocation, and homeostasis have been identified in rice (Goff *et al.*, 2002; Ai *et al.*, 2009; Jia *et al.*, 2011; Sun *et al.*, 2012). To determine whether the improved Pi uptake and increased Pi accumuation in *oscslf6* mutants were associated with the induction of expression of genes encoding Pi transporters, the expression of *OsPT1*, *OsPT2*, *OsPT4*, *OsPT6*, and *OsPT8* were analysed. Under Pi-sufficient conditions, the expression of *OsPT1, OsPT2, OsPT4, and OsPT8* was significantly increased in both shoots and roots of *oscslf6* mutants (Fig. 5B, C). *OsPT6* was up-regulated in roots but was repressed in shoots of *oscslf6* mutants (Fig. 5B,C). These results indicate that enhanced Pi uptake in the shoots and roots of *oscslf6* mutants, which results in increased Pi accumulation at HP levels, are positively correlated with the increased expression of these OsPHT genes in these tissues.

### Knockout of *OsCSLF6* affects the expression of PSR genes

**Fig. 6.**
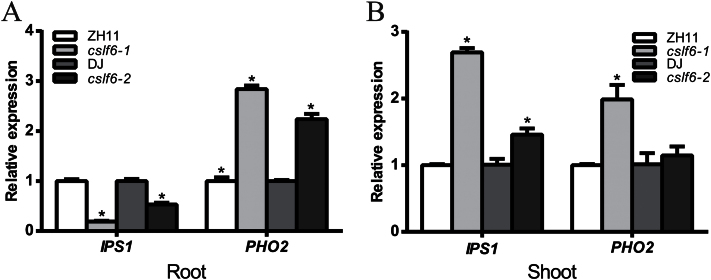
Expression of Pi signalling pathway genes in the WT and *oscslf6* mutants. (A) Expression of *OsIPS1* and *OsPHO2* in roots of WT (ZH11), *oscslf6-1*, WT (DJ), and *oscslf6-2* mutant plants under HP conditions. (B) Expression of *OsIPS1* and *OsPHO2* in shoots of WT (ZH11), *oscslf6-1*, WT (DJ), and *oscslf6-2* mutant plants under HP conditions. Expression was normalized to that of ubiquitin. Error bars indicate SD (*n*=3). Asterisks indicate the significance of differences between WT and *oscslf6* mutant plants as determined by Student’s *t* test: *P ≤0.05.

To test whether the genes related to the Pi starvation-response were also affected by loss-of-function of *OsCSLF6*, the expression of two genes (*OsIPS*1 and *OsPHO2*) were examined by qRT-PCR in *oscslf6* mutants under Pi-sufficient conditions. *OsIPS1* and *OsPHO2* have been shown to be involved in the Pi starvation signalling pathway. Our results show that the expression of *OsIPS1* was significantly increased in shoots but dramatically repressed in roots of *oscslf6* mutants (Fig. 6A, B). The expression of *OsPHO2* was significantly increased in the roots or shoots of *oscslf6* mutants (Fig. 6A, B). These results implied that *OsCSLF6* affects the phosphate starvation signalling pathway in rice.

### Knockout of *OsCSLF6* increases Suc level

**Fig. 7.**
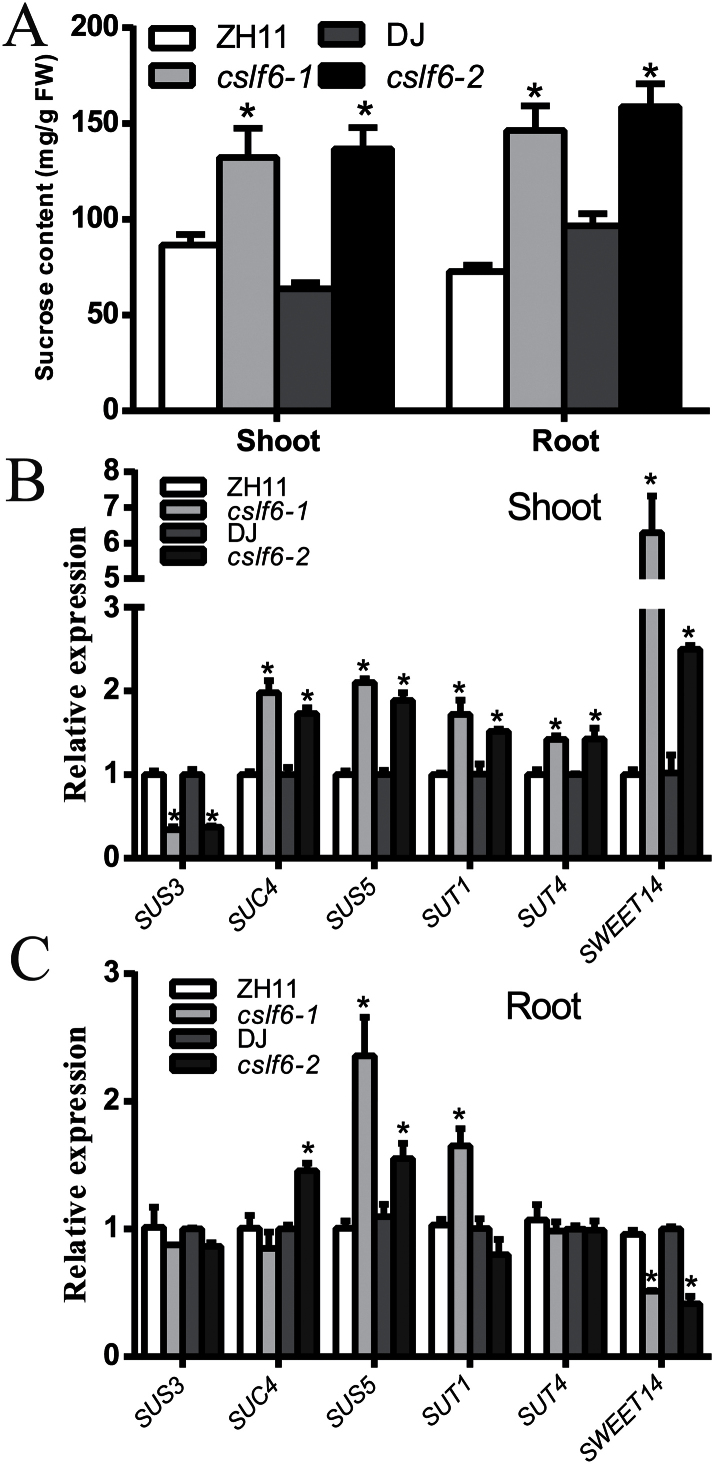
Accumulation of Suc in the *oscslf6* mutants. (A) Soluble sugars were extracted from 12-d-old WT and *oscslf6* mutants. Suc contents were determined by gas chromatography–mass spectrometry. (B–C) Expression of *SUS3*, *SUS4*, *SUT1*, *SUT4*, and *Sweet14* in both shoots (B) and roots (C) of 12-d-old WT and mutants. Expression was normalized to that of ubiquitin. Error bars indicate SD (*n*=3). Asterisks indicate the significance of differences between WT and *oscslf6* mutant plants as determined by Student’s *t* test: *P ≤0.05.

It has been reported that Suc signalling is involved in plant responses to Pi starvation (Chiou and Lin, 2011) and knockout of *OsCSLF6* caused an obvious change in cell wall monosaccharide composition (Vega-Sanchez *et al.*, 2012). The Suc content in the *oscslf6* mutants was then examined. The result showed that the accumulation of Suc in both shoots and roots of both mutants (*oscslf6-1* and *oscslf6-2*) seedlings were much higher than in the WT plants (Fig. 7A). The expression was also examined of Suc synthases (*SUSs*) and Suc transporters (*SUTs*) that are involved in Suc synthesis and transport. QRT-PCR analyses showed that the expression of *SUS4*, *SUS5*, *SUT1*, and *SUT4* was dramatically enhanced although *SUS3* was repressed in shoots of the *oscslf6* mutants (Fig. 7B, C). *OsSweet14*, a gene that functions as Suc transporters in the HEK293T cell line and *Xenopus* oocytes, was also significantly increased in shoots and decreased in roots of the *oscslf6* mutants compared with the WT (Fig. 7B, C) (Chen et al., 2010, 2012). These results demonstrated that knockout of *OsCSLF6* results in increased Suc accumulation in rice, possibly through the activation of *SUS* and *SUT* genes. Together, these results suggest that *OsCSLF6* may have a role in affecting the Pi pathway by changing the level of Suc.

### *Oscslf6* is defective in cell walls

**Fig. 8.**
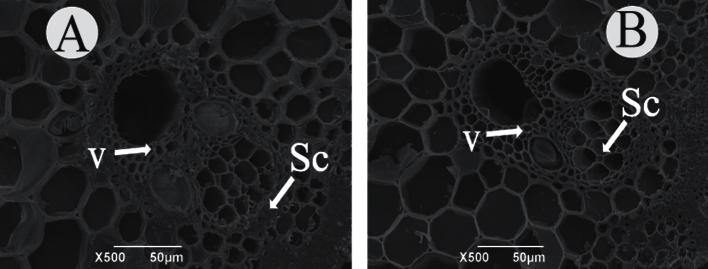
Scanning electron analysis in ZH11 and *oscslf6-1* mutant plants. (A, B) Scanning electron in cross culms sections of ZH11 (A) and *oscslf6-1* (B) plants. Sc, sclerenchyma cells; V, vascular bundles. Bars=50 μm.

Cell wall morphology was also examined with scanning electron microscopy. Scanning electron microscopy observations revealed that the WT sclerenchyma cell walls were heavily thickened and that the cells were nearly completely filled at the mature stages of culms (Fig. 8A), in striking contrast to those of *oscslf6-1* mutant plants (Fig. 8B). To investigate cell wall composition of the *oscslf6* mutants, transverse sections of the culms of the WT and mutant were histochemically stained with Calcofluor solutions. Calcofluor showed much stronger fluorescent signals in the sclerenchyma cells, parenchyma cells, and vascular bundles of the WT than in the *oscslf6-1* mutant (see Supplementary Fig. 4A, B at *JXB* online), demonstrating a significantly high level of cellulose in wild-type plants. This finding is consistent with the scanning electron microscopy observations and indicates that the *oscslf6* mutant is deficient in the cell walls.

### Expression pattern of *OsCSLF6*

For histochemical analysis of the expression pattern of *OsCSLF6*, the promoter (a 1.8-kb fragment upstream of ATG) of *OsCSLF6* was fused to the GUS reporter gene and transformed into WT rice. GUS activity was preferentially detected in the vascular tissues in the tissues examined, such as panicles, leaf lamina joints, root s, and culms (see Supplementary Fig. 3A–I at *JXB* online), and the highest GUS activity was detected in the roots (see Supplementary Fig. 3F–I at *JXB* online). Total RNA was extracted from flag leaves, mature leaves, roots, leaf lamina joints, panicles, and culms, and qRT-PCR analyses were performed. The result showed *OsCSLF6* was expressed in all the organs examined, with expression being lowest in mature leaves and highest in roots (see Supplementary Fig. 3J at *JXB* online), which was consistent with the GUS analysis.

## Discussion

Proteins encoded by the CSLs super-family of genes are known to be involved in the biosynthesis of cell-wall polymers and in polysaccharide biosynthesis (Richmond and Somerville, 2000). Members of the *CSLF* super-family, which are only found in grasses (Hazen *et al.*, 2002), are believed to control the synthesis of mixed-linkage glucan (β-1,3; 1,4, glucan) (Burton *et al.*, 2006). The novel function of *OsCSLF6*, a member of this family, in affecting Pi accumulation and root development, possibly through the alteration of carbon metabolism in rice, is reported here.

### *OsCSLF6* regulates Pi uptake or transport by affecting the expression of PHT genes

The *oscslf6* mutants displayed excessive Pi accumulation and toxic leaf symptoms and growth retardation similar to that of *OsPHR2(O)*, *OsPT2(O)*, and *ospho2* mutants (Liu *et al.*, 2010; Hu *et al.*, 2011). These phenotypes suggest that Pi absorption and translocation may be altered in *oscslf6* mutants. Pi transporters are directly responsible for Pi acquisition and transport in plants (Harrison *et al.*, 2002; Misson *et al.*, 2004; Seo *et al.*, 2008), and plants up-regulate the expression of Pi transporters to enhance Pi uptake and transport efficiency (Liu *et al.*, 1998; Karthikeyan *et al.*, 2002; Xiao *et al.*, 2006; Hu *et al.*, 2011). Our results showed that the expression of *OsPT1/2/4/8* was significantly increased in both roots and shoots of os*cslf6* mutants compared with WT plants, whereas *OsPT6* was dramatically repressed in *oscslf6* shoots under Pi-sufficient conditions. The differential response of PHT genes in both roots and shoots of *oscslf6* mutants suggested that Pi uptake or transport in the *oscslf6* mutant were affected. Increased expression of *OsPT1/2/4/8* in roots may be related to Pi uptake and Pi transport from the roots to the shoots in the *oscslf6* mutants. In addition, the expression of *OsPT6* was dramatically repressed in shoots of *ospho2* which may lead to defective Pi distribution and mobilization (Hu *et al.*, 2011). Similarly, the significantly reduced *OsPT6* expression in *oscslf6* shoots may also result in defective Pi mobilization in these plants. Taken together, our results strongly indicate that *OsCSLF6* is involved in Pi accumulation by affecting the expression of some PHT genes in rice.

### *OsCSLF6* is involved in regulating root development

Root architecture, the spatial configuration of a root system in the soil, has been shown to be important for plant P acquisition (Lynch, 1995). In *Arabidopsis*, plants show dramatic changes in root architecture, including reduced primary root growth and the increased formation of lateral roots and root hairs under low Pi availability (Williamson *et al.*, 2001; López-Bucio *et al.*, 2003). Different from *Arabidopsis*, the elongation of rice primary and adventitious roots are the typical traits stimulated by Pi starvation (Wissuwa, 2003; Zhou *et al.*, 2008a). To investigate whether *OsCSLF6* is involved in this process, the effect of Pi availability on root architecture alteration was analysed in WT and *oscslf6* mutant plants. In our observations, consistent with the over-accumulation of Pi, a significant decrease in primary root length, and adventitious root length and number were observed in *oscslf6* mutants compared with the WT under Pi-sufficient conditions. However, those parameters were alleviated under Pi-deficient conditions. Meanwhile, the Pi content in roots of the *oscslf6* mutants was increased in the *oscslf6* mutants under Pi-sufficient conditions but no significant difference was observed compared with the WT under Pi starvation conditions (Fig. 3F). Taken together, these results indicated that *OsCSLF6* may affect Pi-dependent root architecture by affecting the Pi status in roots.

### *OsCSLF6* may play a role in the balance between carbon metabolism and phosphate accumulation

Increased carbohydrates have been associated in many plant species with low P availability. Over-accumulation of Suc in roots precedes the induction of PSR and the inhibition of Suc biosynthesis or translocation attenuates the plant response to Pi starvation (Hammond and White, 2008, 2011). In *Arabidopsis*, over-expression of *SUC2* resulted in the increased translocation of Suc in the phloem and enhanced sensitivity to Pi starvation (Lei *et al.*, 2011). The authors argued that Suc is a global regulator of P-starvation and elevated levels of Suc can directly alter the expression of a large number of PSI genes (Lei *et al.*, 2011). It has also been reported that over-expression of *ZmPTF1* improves low phosphate tolerance of maize by regulating carbon metabolism (Li *et al.*, 2011). This evidence suggests a role of carbohydrates in communicating the plant responses to low Pi availability. In our results, knockout of *OsCSLF6* increased the Pi content and enhanced the expression of some PSI genes such *OsPT1/2/6/8*. Increased Suc levels were also observed in the *oscslf6* mutants compared with WT plants. Therefore, it is possible that increased Pi accumulation in *oscslf6* mutants result from the alteration of carbon metabolism and carbohydrate allocation.

IPS1 is a PSI gene that negatively affects plant uptake (Franco-Zorrilla *et al.*, 2007) and over-expression of *AtIPS1* results in the increased accumulation of *PHO2* mRNA and reduced shoot Pi content in *Arabidopsis*. *OsIPS1* (Hou *et al.*, 2005) and *OsPHO2* (Hu *et al.*, 2011), the two rice homologue genes to *AtIPS1* and *PHO2*, respectively, function similarly to their *Arabidopsis* counterparts in response to Pi starvation. It was observed that the knockout of *OsCSLF6* suppressed *OsIPS1* in roots but induced *OsIPS1* in shoots, while the expression of *OsPHO2* was increased in both shoots and roots of the *oscslf6* mutants. Similar results were also obtained in the *Arabidopsis pho3* mutant that had a defective Suc transporter 2 (*SUC2*) (Lei *et al.*, 2011). In this mutant, substantially reduced transportation of SUC from the shoot to the root and decreased expression of PSR genes in the root, but increased expression in the shoots, were observed (Lloyd and Zakhleniuk, 2004; Lei *et al.*, 2011). These contrasting expression patterns of PSI genes (*OsIPS1* and *OsPHO2*) may be correlated with the altered accumulation patterns of Suc in the *oscslf6* mutants.

It was also found that the loss of *OsCSLF6* affected the biosynthesis of secondary cell walls and resulted in altered cellulose content (Fig. 8C; see Supplementary Fig. 4A–C at *JXB* online). It is possible that the alteration in cell wall composition or structure affects the expression of PSI genes, such as *OsIPS1* and *OsPHO2*, that are involved in Pi signalling through the plant vasculature. Xylem and phloem provide high-speed pathways for long-distance transportation in plants (Turnbull and Lopez-Cobollo, 2013; Lin *et al.*, 2014). Various kinds of molecules, such as inorganic nutrients, phytohormones, and other metabolites are distributed by the xylem and phloem throughout the plants (Ye, 2002; Lough and Lucas, 2006; Turnbull and Lopez-Cobollo, 2013; Lin *et al.*, 2014). Therefore, the possible effect of altered cell wall composition/structure on Pi assimilation could not be ruled out.

## Supplementary data

Supplementary data can be found at *JXB* online.

Supplementary Fig. S1. Molecular features of *OsCSLF6*.

Supplementary Fig. S2. Phenotypes of *oscslf6-1* mutant plants under high-nitrogen and nitrogen-deficient conditions.

Supplementary Fig. S3. *OsCSLF6* expression profile.

Supplementary Fig. S4. Staining of cellulose in ZH11 and *oscslf6-1* mutant plants.

Supplementary Table S1. Primers used in this study.

Supplementary Table S2. Primers for real-time PCR analysis.

Supplementary Data
