# Supplementary material for: Interaction between carbon metabolism and phosphate accumulation is revealed by a mutation of a cellulose synthase-like protein, CSLF6
Source: J Exp Bot. 2015 Mar 4;66(9):2557–67. doi: 10.1093/jxb/erv050 (PMC4986868; doi:10.1093/jxb/erv050)
Supplement: Supplementary Data [file supp_66_9_2557__index.html]

Interaction between carbon metabolism and phosphate accumulation is revealed by a mutation of a cellulose synthase-like protein, CSLF6 — Interaction between carbon metabolism and phosphate accumulation is revealed by a mutation of a cellulose synthase-like protein, CSLF6 — Interaction between carbon metabolism and phosphate accumulation is revealed by a mutation of a cellulose synthase-like protein, CSLF6 — Supplementary Data 

# Interaction between carbon metabolism and phosphate accumulation is revealed by a mutation of a cellulose synthase-like protein, CSLF6

## Supplementary Data

Data files

**Files in this Data Supplement:**

- Supplementary Data - Supplementary Data
