# Supplementary material for: Interaction between carbon metabolism and phosphate accumulation is revealed by a mutation of a cellulose synthase-like protein, CSLF6
Source: J Exp Bot. 2015 Mar 4;66(9):2557–67. doi: 10.1093/jxb/erv050 (PMC4986868; doi:10.1093/jxb/erv050)
Supplement: Supplementary Data [file supp_erv050_jexbot141499_file001.pdf]

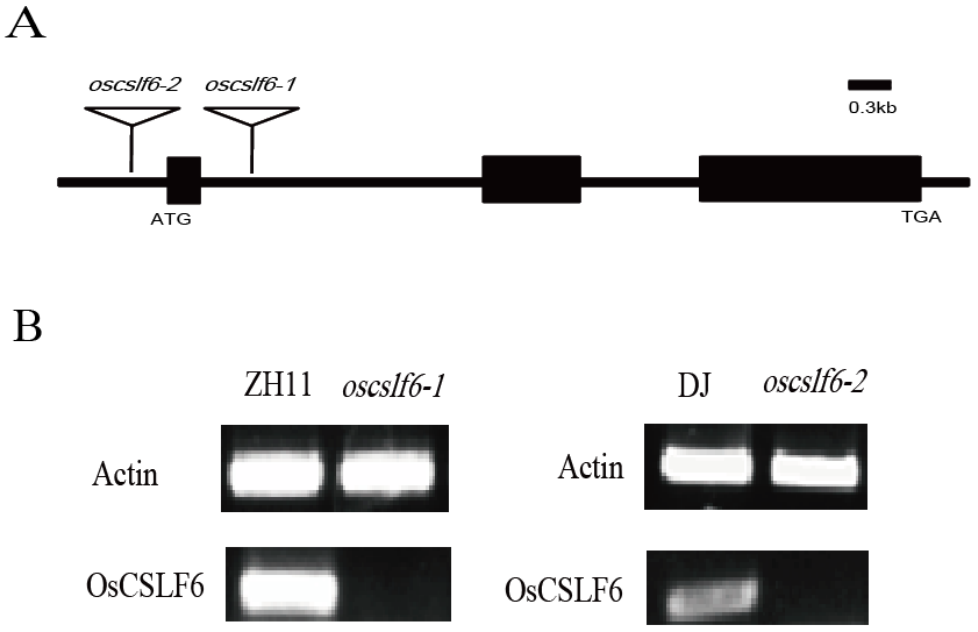

Figure S1. Molecular features of *OsCSLF6*. (A) Structure of *OsCSLF6* and T-DNA insertion site. Three exons (filled boxes) and two introns (lines between the filled boxes) are shown. T-DNA insertion sites of *oscslf6-1* and *oscslf6-2* were located in the first intron and 204bp upstream of ATG. (B) RT-PCR analyses of *OsCSLF6* expression levels in the ZH11, *oscslf6-1*, DJ, *oscslf6-2* plants. Total RNA was extracted from 9-d-seedling. Actin was used as a control.

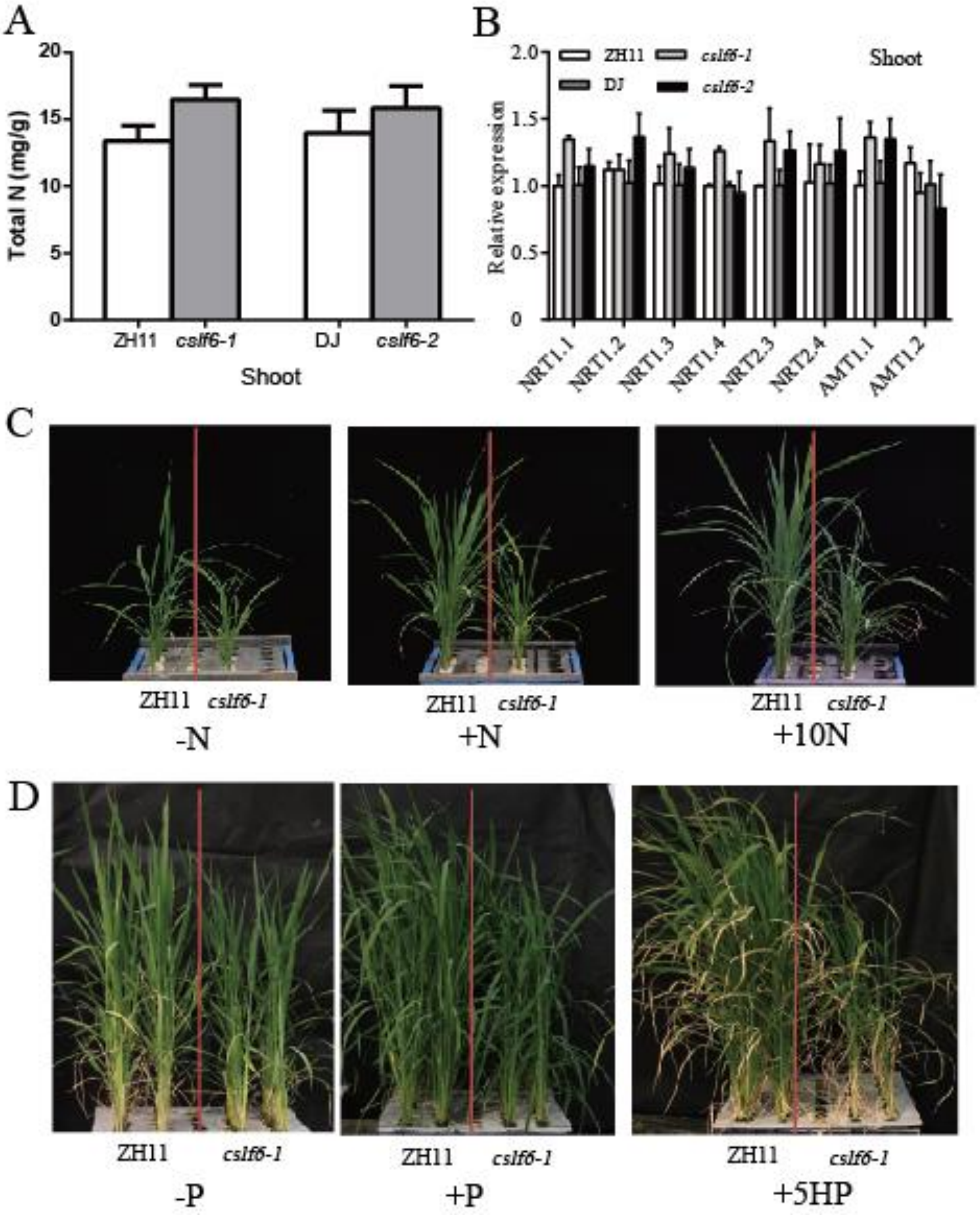

Figure S2. Phenotypes of *oscslf-1* mutants under different N conditions. (A) Shoot total N content in ZH11 and *oscslf6-1* mutant plants. (B) Expression of Nitrogen related transporter genes in shoots of ZH11 and *oscslf6-1* mutant plants. Error bars indicate SD (n=3). (C) Phenotypes of ZH11 and *oscslf6-1* mutant plants under –N (no N), +N (1.25 mM) and +10N (12.5 mM NH_4NO_3) conditions. (D) Phenotypes of ZH11 and *oscslf6-1* mutant plants under –Pi (no Pi), +P (0.32 mM) and +5 Pi (1.6 mM NaH_2PO_4) condition.

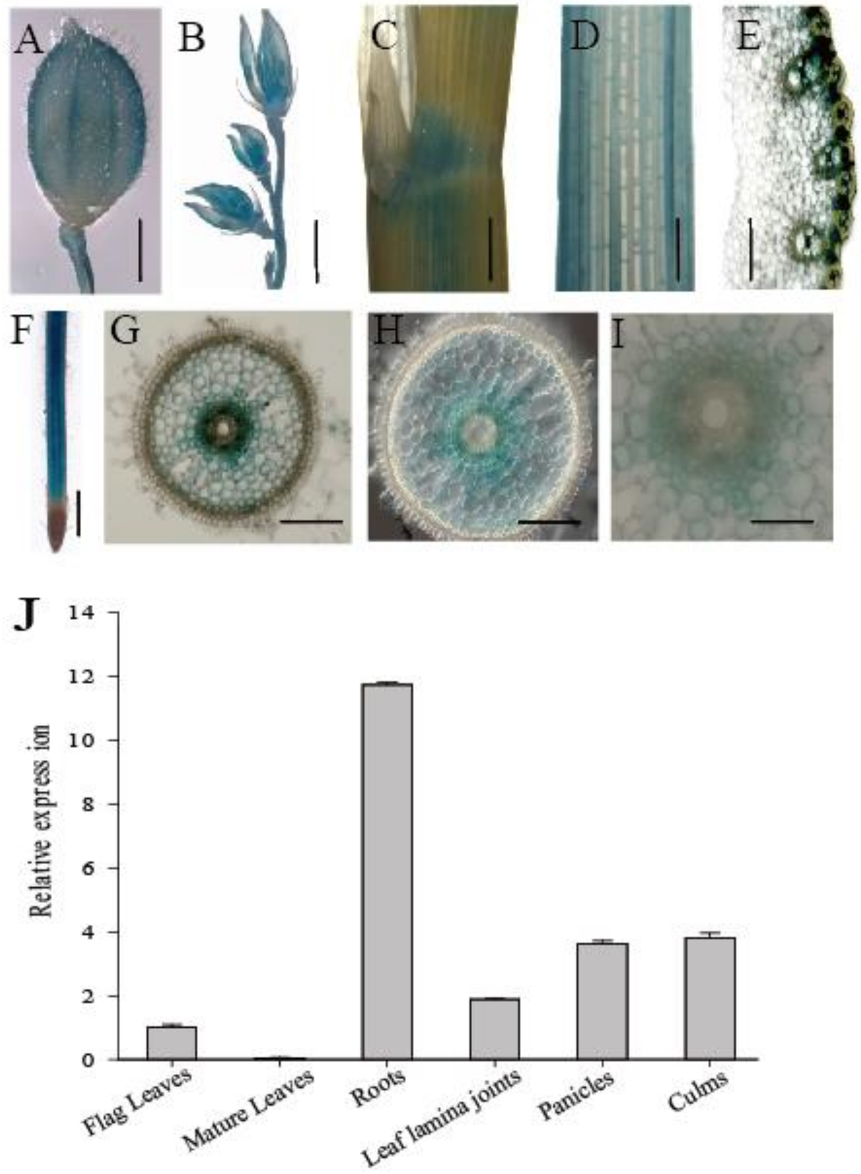

Figure S3**.** *OsCSLF6* expression profile. (A–F) GUS staining of *OsCSLF6* PRO::GUS transgenic plant: Grain husks (A); young panicles (B); Leaf lamina joints (C); Culms (D); Cross-section of culms (E); Roots (F). (G-I) Cross-section of roots. Bars=2 mm (A-B, D), 4 mm (C), 1 mm (F-G), 500 μm (H), 300 μm (E, I). (J) Relative *OsCSLF6* expression levels in the Flag leaves, Leaves, Roots, Leaf lamina joints, panicles, and culms. Error bars indicate SD (n=3).

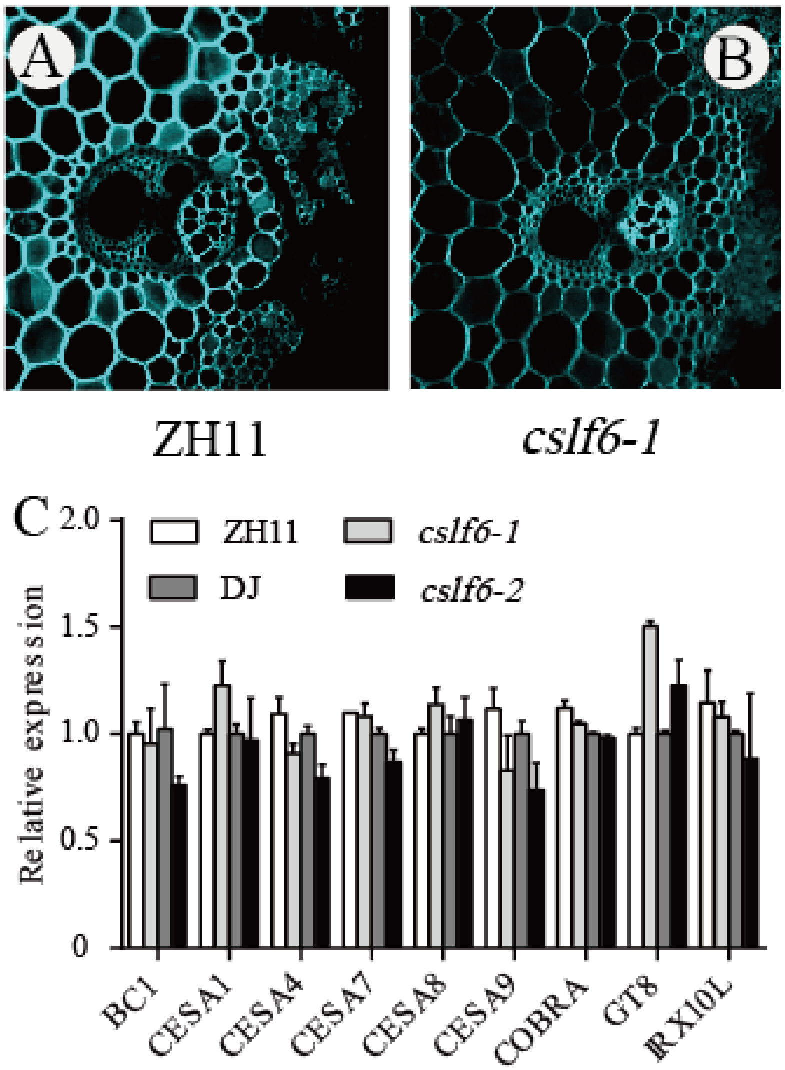

Figure S4. Cellulose staining in transverse culms sections of ZH11 (A) and *oscslf6-1* (B) plants. (C) Expression of cell wall synthesis related genes of ZH11, *oscslf6-1*, DJ and *oscslf6-2* mutant plants. Error bars indicate SD (n=3).

Table S1. Primers used in this study

Primer name	Forward primer (5'–3')	Reverse primer (5'–3')
CSLF6, Promoter-Gus	attB1-CAATCCCTTTCGGGTTGGTTG	attB2-CATTGCTAATGCCTTTGCCTCTC
CSLF6, RT-PCR	CATCATCATCCTCGTCAACATC	GGTGAAGTCGTACTTCTTGGTG
Actin	TTCCTACATCGCCCTGGACT	AGCCTTGGCAATCCACATCT

Table S2. Primers for real-time PCR analysis

Gene	Forward primer (5'–3')	Reverse primer (5'–3')
OsIPS1	CTAAGGTAGGGCAACTTGTATC	TTATTAGAGCAAGGACCGAAAC
OsPHO2	TTTTACACAAGCCACCAAAGC	TCACGAGCATGTCCAACAA
OsCSLF6	CGCTACTGCTCCATCTACCC	GGCACGGTGGTGTAGAAGAT
OsPT1	CGCTTCCGTACGAGTGGTAGT	GGTTCTTTCAAATCCAGGGAAA
OsPT2	GACGAGACCGCCCAAGAAG	TTTTCAGTCACTCACGTCGAGAC
OsPT4	TTCTGCTAGTGTACCAAACAAAATTACA	CTAAGTGGCATTTATAATATCAACAGTA ACC
OsPT6	CCGCCCCTGCAAACTGTA	CAACTGGCGGTTTCTTCGAT
OsPT8	AGAAGGCAAAAGAAATGTGTGTTAAAT	AAAATGTATTCGTGCCAAATTGCT
OsBC1	ACCCCAACCTCAACAACGTCA	AACATGCCGGTGTCGTTGAT
OsCESA1	TTGACTTGCACGATCGATACG	TCCCACATAAACTGGACCCTG
OsCESA4	GTTCGATGGCATTGATCGCA	CCACATAAACCGGACCTTGGA
OsCESA7	CCGGATGGATGATTCTTGTTG	CCCCCAAAACACTTTTATCCC
OsCESA9	AGGCCATCCATGTCATCAGCT	TTGAACCCCGTTAGGATGTCC
OsCOBRA	ACGATTTGCTCATGCAAGCTGGTC	TCCGGAGGTGGCATGACACAATTA
OsGT8	AGATTGATTCGTTAGGCTTC	AAATGAAACCTTTATCCTATCCA
OsGUX1L	ACTGTAACTGGAATAACC	AAGACAATAGTGCTGAAG
OsNRT1.1	CGAGGTTGGTGCATTTTGTG	GCCGTGGTGTTCTCTTTTTTT
OsNRT1.2	CAATCTGTAATGCAGGGTTAACTGTT	AGCATCCTCAAGACCACCAAA
OsNRT1.3	GAGGTTTGGGTTTTTGAGGTAGTG	CAGGAGATTGAAGCTAGCATCATATC
OsNRT1.4	AATGATCCCTGATTAGGTCAAGTCA	CCAAATACCACTACTCTTGCATCCT
OsNRT2.3	ACGGAGACCGGGATCAAGTA	CCCACTGCGGGAAGTAGATG
OsNRT2.4	ACGAAGCTGGTGGAAGAAGAAG	ACGACGCCATCGCCATA
OSAMT1.1	TCTCTTCTACGGGCTCAAGAAGC	CAAATTTATGACGTGACGATCGAGA
OSAMT1.2	GATCTACGGCGAGTCGGGCACGA	TTCCATCTCTGTCGAGGTCGAGACG
Ubiquitin	AACCAGCTGAGGCCCAAGA	ACGATTGATTTAACCAGTCCATGA
